# Free Salivary Amino Acid Profile in Breast Cancer: Clinicopathological and Molecular Biological Features

**DOI:** 10.3390/cimb46060336

**Published:** 2024-06-05

**Authors:** Lyudmila V. Bel’skaya, Elena A. Sarf, Denis V. Solomatin

**Affiliations:** 1 Biochemistry Research Laboratory, Omsk State Pedagogical University, 644099 Omsk, Russia; sarf_ea@omgpu.ru; 2 Department of Mathematics and Mathematics Teaching Methods, Omsk State Pedagogical University, 644099 Omsk, Russia; solomatin_dv@omgpu.ru

**Keywords:** saliva, metabolome, amino acids, breast cancer, breast benign lesion, molecular biological subtype, diagnostics

## Abstract

The study of salivary amino acid profiles has attracted the attention of researchers, since amino acids are actively involved in most metabolic processes, including breast cancer. In this study, we analyzed the amino acid profile of saliva in a sample including all molecular biological subtypes of breast cancer to obtain a more complete picture and evaluate the potential utility of individual amino acids or their combinations for diagnostic purposes. This study included 116 patients with breast cancer, 24 patients with benign breast disease, and 25 healthy controls. From all patients, strictly before the start of treatment, saliva samples were collected, and the quantitative content of 26 amino acids was determined. Statistically significant differences between the three groups are shown in the content of Asp, Gly, Leu + Ile, Orn, Phe, Pro, Thr, and Tyr. To differentiate the three groups from each other, a decision tree was built. To construct it, we selected those amino acids for which the change in concentrations in the subgroups was multidirectional (GABA, Hyl, Arg, His, Pro, and Car). For the first time, it is shown that the amino acid profile of saliva depends on the molecular biological subtype of breast cancer. The most significant differences are shown for the luminal B HER2-positive and TNBC subgroups. In our opinion, it is critically important to consider the molecular biological subtype of breast cancer when searching for potential diagnostic markers.

## 1. Introduction

Saliva is a unique biological fluid that contains a huge amount of information about the state of the human body [1]. In recent decades, it was discovered that saliva can be used in medical examinations [2,3]. Saliva is a promising tool for diagnosing and monitoring diseases, as well as guiding treatment [4,5,6]. Saliva has a great potential for diagnosing a wide range of diseases, including cancer [7,8]. One of the key advantages of saliva diagnostics in detecting cancer is its non-invasiveness, as saliva collection is a simple and painless process that does not require any special equipment or experience. Saliva collection can be easily performed in a clinical setting or even at home, making it convenient for patients [9]. Saliva diagnostics also offer the advantage of the early detection of diseases [10,11].

The development of metabolomics as one of the components of the new direction of “Salivaomics” has brought saliva research to a qualitatively new level [12]. The metabolome is the complete set of low molecular weight metabolites, including metabolic intermediates such as carbohydrates, lipids, amino acids, nucleic acids, hormones, and other signaling molecules [2]. Metabolomic analyses can be performed as targeted or untargeted. Targeted metabolomics approaches analyze specific metabolites or associated pathways that are candidate biomarkers [13]. The goal of untargeted metabolomic studies is to measure the widest possible range of metabolites in a sample and search for new biomarkers to identify the phenotype [14]. The study of the salivary metabolome in cancer, including breast cancer, has been a popular scientific area in recent years [15,16,17,18,19,20,21,22].

The study of amino acids, as one of the components of metabolomics, in saliva has attracted special attention from researchers, since amino acids take an active part in most metabolic processes, including glycolysis (Gln, Gly, and Ser), regulate the production of reactive oxygen species, etc. [23]. However, the works of different authors identified different amino acids, the changes in which were statistically significant in breast cancer. Thus, Sugimoto et al. found eight amino acids in a number of metabolites for the diagnosis of breast cancer (Lys, Thr, Leu + Ile, Glu, Tyr, Val, and Gly) [24]. Cheng et al. analyzed 17 amino acids to distinguish stage I–II breast cancer from healthy controls and proposed a comprehensive index for the detection of early breast cancer, which included only three amino acids: Pro, Thr, and His [25]. Zhong et al. [26] identified two potentially informative amino acids for breast cancer diagnosis: Phe and His. Murata et al. emphasized the four amino acids Leu, Gln, Ile, and Ser for the diagnosis of breast cancer [27]. It should be noted that the authors identified different amino acids that are important in the diagnosis of breast cancer. Thus, in three studies, Leu + Ile are common, but there is no justification for the choice of these particular amino acids from the point of view of the biochemistry of the ongoing processes, which has yet to be conducted.

In this study, we analyzed the amino acid profile of saliva in a sample including all molecular biological subtypes of breast cancer to obtain a more complete picture and evaluate the potential utility of individual amino acids or their combinations for diagnostic purposes.

## 2. Materials and Methods

### 2.1. Study Design

This study included 116 patients with breast cancer (main group; age, 56.6 ± 2.3 years); 24 patients with non-malignant pathologies of the mammary glands (comparison group; age, 47.0 ± 4.5 years); and 25 volunteers selected as healthy controls (control group; age, 39.7 ± 4.1 years). Patients of the main group and the comparison group were recruited from the admission department of the Clinical Oncology Dispensary in Omsk. Patients with breast cancer and non-malignant breast pathologies were hospitalized for surgical treatment. Only after histological verification were patients assigned to the appropriate group (BC or BBL). Some patients with a confirmed diagnosis of breast cancer were hospitalized for the first course of chemotherapy. Volunteers in the control group were active blood donors and underwent a full medical examination. All volunteers, based on the results of mammography and ultrasound examination, were confirmed to have no pathologies of the mammary glands.

Inclusion criteria: Female gender; patient aged 30–60 years; absence of any treatment at the time of the study, including surgery, chemotherapy or radiation; and absence of signs of active infection (including purulent processes). All participants were examined by a dentist and had good oral hygiene. Exclusion criteria: lack of histological verification of the diagnosis.

### 2.2. Collection of Saliva Samples

Saliva samples were collected during hospitalization strictly before the start of treatment. Samples were collected in sterile polypropylene centrifuge tubes with a screw cap in a volume of two ml. Saliva samples were collected by spitting without additional stimulation in the interval of 8–10 a.m., the time of maximum saliva secretion, on an empty stomach after preliminarily rinsing the mouth with water. We did not find significant differences in the salivary flow rate in the studied groups, so they are not shown in the tables below.

Immediately after collection, samples were centrifuged at 10,000× *g* for 10 min (CLb-16, Moscow, Russia); 1 mL of the upper layer was taken; transferred to Eppendorf tubes; and stored in a freezer at −80 °C until analysis.

### 2.3. Determination of the Amino Acid Composition of Saliva

In all saliva samples, we determined the content of 26 amino acids (1-MH, GABA, Hyl, Ala, Arg, Asn, Asp, Car, Cit, Glu, Gln, Gly, His, Hcit, Leu + Ile, Met, Orn, Phe, Pro, Sar, Ser, Thr, t4HYP, Trp, Tyr, and Val). The volume of the aliquot for analysis was 40 μL, and, in each case, three parallel determinations were carried out.

Samples were analyzed using high-performance liquid chromatography on a 1260 Infinity II chromatograph (Agilent, Santa Clara, CA, USA) with detection on a 6460 Triple Quad mass spectrometer (Agilent, Santa Clara, CA, USA). The samples were separated by liquid chromatography using an Agilent Zorbax Eclipse XDB-C18 2.1 × 100 mm column with a sorbent diameter of 1.8 μm (Agilent, Santa Clara, CA, USA). To analyze the test compounds in samples, an HPLC method with mass spectrometric detection in the monitoring mode of selected reactions was developed. The internal standard method was used to back-calculate concentrations. Alanine-d4 was used as an internal standard. The detection of amino acids was performed in the mode of monitoring selected reactions in accordance with the list of SRM transitions for each of the analyzed amino acids. The dependence of the concentration of amino acids on the ratio of the peak areas of amino acids to the peak area of the internal standard was preliminarily calculated. To construct a calibration scale, at least six solutions of individual amino acids were used (Jasem, Istanbul, Turkey), with concentrations selected in accordance with the content of amino acids in saliva based on the results of a preliminary determination. The automatic integration of chromatograms was used using the Quantitative Quant-my-way software (MassHunter Workstation Quantitative Analysis B.09.00) (Agilent, Santa Clara, CA, USA).

### 2.4. Determination of the Expression of the Receptors for Estrogen, Progesterone, HER2, and Ki-67

The Allred Scoring Guideline was used to assess the level of expression of estrogen receptors (ERs), progesterone receptors (PRs), and HER2 [28]. The level of expression of estrogen, progesterone, and HER2 receptors was assigned to one of four categories (−, +, ++, or +++), in accordance with the ASCO/CAP recommendations [29]. Ki-67 expression was determined as part of a standard breast cancer panel, according to the manufacturer’s protocol [30]. The cut-off value for Ki-67 was defined as 14% (low Ki-67) and 40% (high Ki-67). According to the obtained values, breast cancer was classified into five groups: triple negative breast cancer (TNBC), luminal A-like, luminal B-like (HER2-negative), luminal B-like (HER2-positive), and HER2-enriched (non-luminal).

### 2.5. Statistical Analysis

A statistical analysis was performed using Statistica 10.0 (StatSoft) programs using a nonparametric method. When comparing two groups, we used the Mann–Whitney test; when comparing three groups or more, we used the Kruskal–Wallis test. The sample was described using the median (Me) and interquartile range in the form of the 25th and 75th percentiles [LQ; UQ]. Differences were considered statistically significant at *p* < 0.05.

A principal component analysis (PCA) was performed using the PCA program in R. PCA results are presented in the form of factor planes and corresponding correlation circles. In each case, the figures show only the first two principal components (PC1 and PC2). The color of the arrows on the correlation circle changes from blue (weak correlation) to red (strong correlation), as shown on the color bar. The orientation of the arrows characterizes positive and negative correlations (for the first principal component, we analyzed the location of the arrows relative to the vertical axis; for the second principal component, it was analyzed relative to the horizontal axis). The significance of the correlation is determined by the correlation coefficient (r): strong—r = ±0.700 to ±1.00, medium—r = ±0.300 to ±0.699, and weak—r = 0.00 to ±0.299.

To construct classification trees, the exhaustive search method for one-dimensional branches CART (Classification and Regression Tree) was used (Statistica 10.0, StatSoft). In the diagrams, ID is the number of the vertex, N is the number of objects directed along this branch, branching conditions are indicated near each vertex, and the diagram inside each vertex shows the classification result: if all the observations are classified correctly, then the column corresponding to the predicted class will be high, and the rest are small.

## 3. Results

This study included three groups: breast cancer (BC), breast benign lesion (BBL), and healthy controls (HCs). The structure of the breast cancer subgroup is shown in Table 1.

In the first stage, we tested the effects of age, BMI, and menopausal status on salivary amino acid levels. There were no statistically significant differences between subgroups based on age and BMI. For patients with and without menopause, differences in Arg content are shown; in the subgroup without menopause, Arg content is higher by 42.0% (*p* = 0.0163). Because differences in Arg levels were not statistically significant in further subgroup comparisons, we did not consider menopausal status in the calculations.

### 3.1. Features of the Amino Acid Profile of Saliva in Breast Cancer in Comparison with Non-Malignant Breast Pathologies and Healthy Controls

The quantitative content of 26 amino acids was determined in the saliva of the studied groups (Table 2). Statistically significant differences between groups are shown in the content of Asp, Gly, Leu + Ile, Orn, Phe, Pro, Thr and Tyr (Table 2).

**Figure 1:**
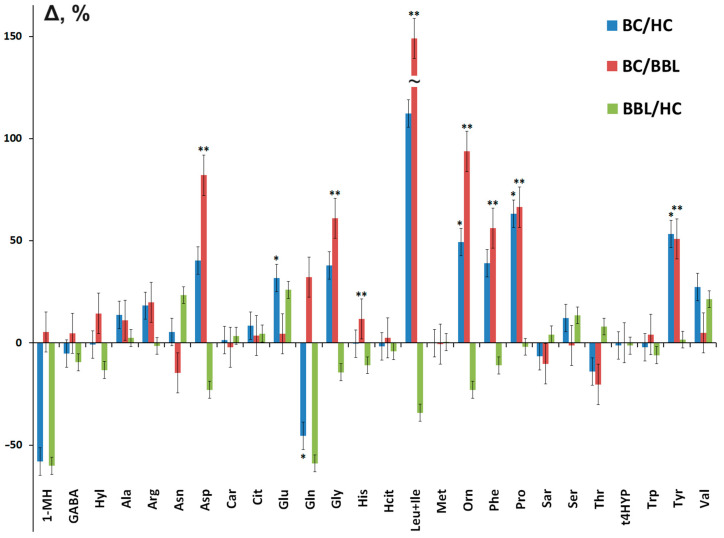
Relative change in the concentration of individual amino acids in the group of patients with breast cancer compared with healthy controls (BC/HCs), compared with the breast benign lesion (BC/BBL) group, as well as in the group of breast benign lesion compared with healthy controls (BBL/HCs). *—the differences between BC and HC are statistically significant, **—the differences between BC and BBL are statistically significant, *p* < 0.05.

At the same time, for Asp, Gly, Leu + Ile, Orn, Phe, Pro, and Tyr, the concentration in saliva in breast cancer increases both compared to the BBL subgroup and the healthy controls, while the concentration in the BBL subgroup decreases compared to the healthy controls (Figure 1). An inverse relationship is shown only for Thr, whereby the concentration in cancer decreases, and in the BBL subgroup, it increases compared to the healthy controls.

**Figure 2:**
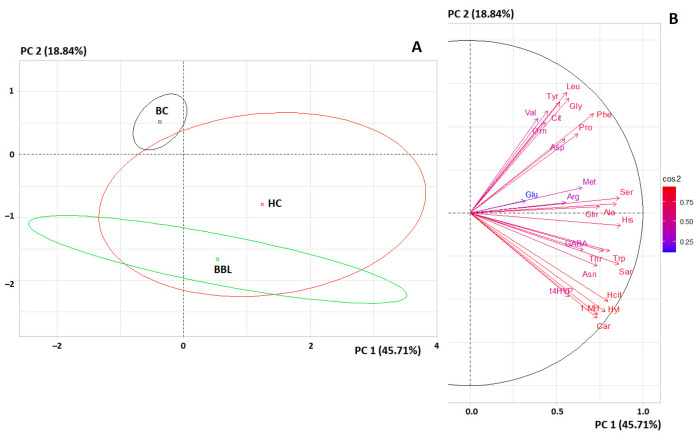
(**A**) Individuals factor map (PCA); (**B**) Variables factor map to separate three groups (BC, BBL, and HC).

The principal component analysis (PCA) showed that the separation of three groups (BC, BBL, and HC) was statistically significant (*p* = 0.0478) (Figure 2A). It can be seen that the separation of the subgroups of breast cancer patients and the BBL subgroup was complete, while the subgroups of the BBL subgroup and the healthy controls were not completely separated from each other. The greatest contribution to the separation of subgroups by the first principal component was made by His (r = 0.8721), Ser (r = 0.8643), Sar (r = 0.8603), Ala (r = 0.8471), and Trp (r = 0.8071). For the second principal component, the largest contributions were made by Leu + Ile (r = 0.6984), Gly (r = 0.6654), Tyr (r = 0.6410), and Car (r = −0.6074) (Figure 2B). It should be noted that the contribution of individual amino acids to the separation of subgroups is approximately equal; it is not possible to identify amino acids that clearly allow the subgroups to be differentiated from each other (Figure 2B).

**Figure 3:**
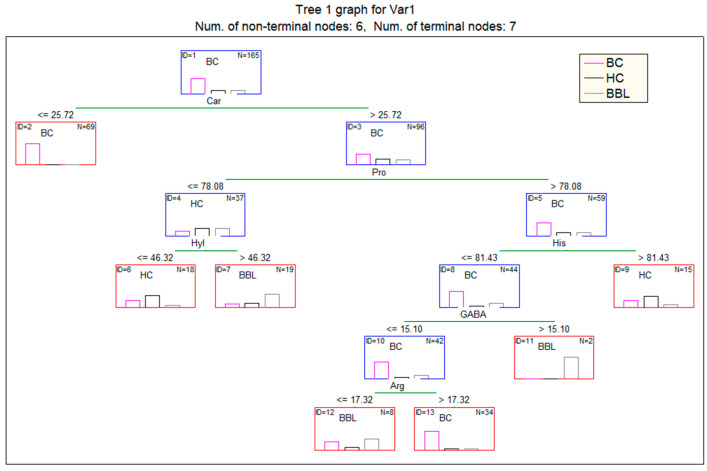
Classification tree for assigning patients to the classes “Breast cancer (BC)/Breast Benign Lesion (BBL)/Healthy Control (HC)”, according to AA values.

At the next stage, we built a decision tree to differentiate the three groups from each other (Figure 3). It is interesting that for constructing the tree, the algorithm did not select those amino acids for which the difference in content between the subgroups was statistically significant (Table 2), but it did for those in which the change in concentrations in the subgroups was multidirectional (GABA, Hyl, Arg, His, Pro, and Car). Apparently, the multidirectional nature of changes in amino acid concentrations in the groups was important for the differentiation of groups.

Table 3 shows the results of classification into three classes: BC, BBL, and HC. It is shown that out of 116 patients with breast cancer, 99 were classified correctly, 17 patients received a false negative result, and 4 patients received a false positive classification result (Table 3). Thus, the sensitivity was 85.3% and specificity 72.0%. The accuracy of BBL detection was 75.0%.

### 3.2. Effect of Breast Cancer Stage on Salivary Amino Acid Profile

Table 4 shows the relative change in amino acid concentrations compared to healthy controls for early and advanced breast cancer. The change in amino acid concentrations was unidirectional, with the exception of two amino acids, for which the differences between early and advanced stages of breast cancer were statistically significant—Thr (*p* = 0.0195) and t4HYP (*p* = 0.0417) (Table 4, Table S1). Amino acids, the differences of which with the control group were statistically significant, differ for early stages and common ones. Thus, for the early stages, the concentration of Gln (*p* = 0.0155) and Glu (*p* = 0.0280) increased statistically significantly, and the concentration of Thr decreased (*p* = 0.0404). For common stages, the concentration of Leu + Ile (*p* = 0.0204) and Phe (*p* = 0.0352) increased. In both cases, an increase in the concentration of Orn (*p* = 0.0128 and *p* = 0.0138) and Tyr (*p* = 0.0197 and *p* = 0.0417) was noted.

Compared with BBL, in breast cancer, both at early and advanced stages, the concentration of Asp (*p* = 0.0289 and *p* = 0.0018), Gly (*p* = 0.0194 and *p* = 0.0158), Leu + Ile (*p* = 0.0114 and *p* = 0.0001), Orn (*p* = 0.0012 and *p* = 0.0024), Phe (*p* < 0.0001), Pro (*p* = 0.0004 and *p* = 0.0036), and Tyr (*p* = 0.0004 and *p* = 0.0023) increased. A distinctive feature of the early stages of breast cancer was a decrease in Thr concentration compared to BBL (*p* = 0.0138) (Table 5).

### 3.3. The Influence of Lymph Node Involvement Status on the Amino Acid Profile of Saliva

When analyzing the influence of the status of lymph node damage, it was shown that with status N_0_, breast cancer patients differed from healthy controls in the content of Glu (*p* = 0.0339), Gln (*p* = 0.0304), Orn (*p* = 0.0139), Thr (*p* = 0.0494), and Tyr (*p* = 0.0186) (Table 4, Table S2). Other differences were found for N_1–3_ status: Leu + Ile (*p* = 0.0220), Orn (*p* = 0.0126), Pro (*p* = 0.0370), and Tyr (*p* = 0.0357). When comparing breast cancer patients with BBL for N_0_ status, differences were observed in the content of Asp (*p* = 0.0471), Gly (*p* = 0.0424), His (*p* = 0.0358), Leu + Ile (*p* = 0.0260), Orn (*p* = 0.0012), Phe (*p* < 0.0001), Pro (*p* = 0.0018), Thr (*p* = 0.0248), and Tyr (*p* = 0.0004). The same differences remained for N_1–3_ status with the exception of Thr (*p* = 0.3545) (Table 5).

### 3.4. The Influence of the Degree of Tumor Differentiation on the Amino Acid Profile of Saliva

When comparing breast cancer groups of different degrees of differentiation (G I+II vs. G III), it was shown that the content of Asn (*p* = 0.0143) and t4HYP (*p* = 0.0071) increased, while the content of Glu (*p* = 0.0101) and Phe (*p* = 0.0499) decreased with a low degree of tumor differentiation (Table S3). Compared with the healthy controls, at G I + II, there was a statistically significant decrease in the content of Gln (*p* = 0.0226), as well as an increase in the content of Phe (*p* = 0.0344), Pro (*p* = 0.0377), and Tyr (*p* = 0.0079). With a low degree of tumor differentiation, differences with the healthy controls in amino acid content decreased (Table 4). If we compare it with the BBL subgroup, then for G I + II, more differences are also seen, in particular, the content of Gly (*p* = 0.0046) and His (*p* = 0.0084) increased (Table 5).

### 3.5. The Influence of the Expression Status of Estrogen, Progesterone, HER2 Receptors, and the Proliferative Activity Index on the Amino Acid Profile of Saliva

There were no statistically significant differences in the content of individual amino acids between the subgroups with a positive and negative expression status of estrogen and progesterone. However, compared with the healthy controls, there was a statistically significant increase in Glu content (*p* = 0.0361) and a decrease in Gln content (*p* = 0.0248) for the subgroup of estrogen-positive breast cancer (Table 4, Tables S4–S7). The subgroup of progesterone-negative breast cancer differed from the healthy controls in the content of Leu + Ile (*p* = 0.0232) and Pro (*p* = 0.0366). Compared with the BBL subgroup, no differences in the amino acid profile depending on the status of estrogen and progesterone receptors were identified (Table 5).

**Figure 4:**
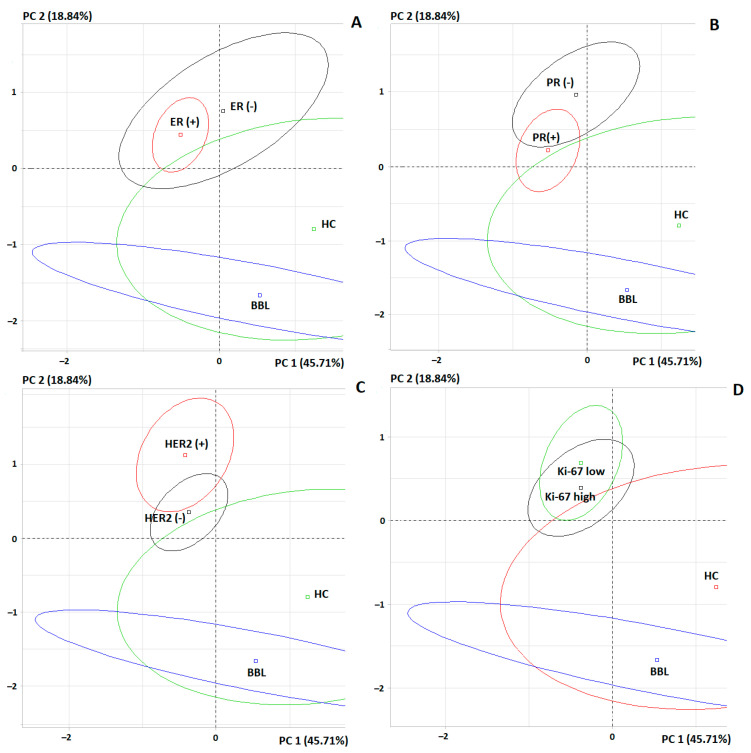
Individuals factor map (PCA) for a comparison of 4 subgroups: (**A**) estrogen receptor expression status, (**B**) progesterone receptor expression status, (**C**) HER2 receptor expression status, (**D**) proliferative activity index (Ki-67).

The PCA method showed that the differences between the subgroups of estrogen-positive and estrogen-negative breast cancer were not separated on the factor diagram (*p* = 0.8366), while the differences with the BBL and HC subgroups were significant in both cases (*p* < 0.0001) (Figure 4A). For progesterone receptors, there was a partial separation of subgroups in the factor diagram (*p* = 0.2025) (Figure 4B). It should be noted that the differences were more pronounced for the progesterone-negative than for the progesterone-positive breast cancer subgroup.

Depending on the HER2 expression status, differences were observed in the content of Asp (*p* = 0.0326), Leu + Ile (*p* = 0.0237), Orn (*p* = 0.0188), and Phe (*p* = 0.0393). Levels of these amino acids were higher in the HER2-positive breast cancer subgroup. In addition to the listed amino acids, when compared with the healthy controls, the HER2-positive breast cancer subgroup had significantly higher levels of Gly (*p* = 0.0261) and Pro (*p* = 0.0394) (Table 4). When compared with non-malignant breast pathologies, the HER2-positive breast cancer subgroup also had increased His levels (*p* = 0.0029) (Table 5). According to the results of the PCA analysis, it was found that it was the subgroup of HER2-positive breast cancer that was significantly different from the BBL and HC subgroups (Figure 4C).

According to the value of the index of proliferative activity, Ki-67 differences were detected for two amino acids: the Asn content increased with a high Ki-67 (*p* = 0.0143), while the Glu content decreased (*p* = 0.0052). With a low Ki-67, compared with the healthy controls, the content of Gln decreased maximally (*p* = 0.0234), and the content of Pro (*p* = 0.0445) and Tyr (*p* = 0.0088) increased. In general, the differences between subgroups with different proliferative activity indexes were practically not pronounced (*p* = 0.6092) (Figure 4D).

### 3.6. The Influence of the Molecular Biological Subtype of Breast Cancer on the Amino Acid Profile of Saliva

Table 6 shows the relative change in amino acid concentrations that differ among the different molecular biological subtypes of breast cancer. It was shown that the luminal B HER2-positive subtype of breast cancer differed the most in amino acid profile: for Ala, Asp, Leu + Ile, Orn, and Trp, a significant decrease in content was shown for all molecular biological subtypes except TNBC (Table 6). Differences in the content of His (*p* = 0.0154), Phe (*p* = 0.0114), and Tyr (*p* = 0.0059) were shown between the luminal B HER2-positive and negative subtypes (Table S8). However, the concentration of amino acids was higher in the HER2-positive breast cancer subgroup. Differences between the luminal B HER2-positive and non-luminal breast cancer subtypes were shown in the levels of Cit (*p* = 0.0318), His (*p* = 0.0192), and Tyr (*p* = 0.0055).

**Figure 5:**
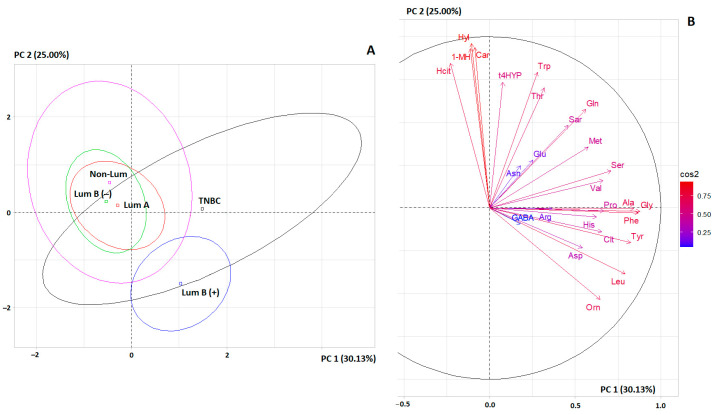
(**A**) Individuals factor map (PCA); (**B**) variables factor map for separation of molecular biological subtypes of breast cancer.

Differences between subgroups were analyzed by PCA (Figure 5A). It can be seen that the amino acid profile differed significantly between the luminal B HER2-positive and TNBC subgroups, despite the fact that there were no differences in individual amino acids between these subgroups. On the other hand, the remaining subgroups were not separated on the factor diagram (Figure 5B), despite the fact that differences between these subgroups were shown in individual amino acids (Table 6). The contribution of most amino acids to the subgroup separation was high; only four amino acids (GABA, Arg, Glu, and Asn) showed a low correlation coefficient (Figure 5B). Thus, subgroups with different molecular biological subtypes differed from the healthy controls more in the combination of amino acids rather than in individual amino acids.

The greatest contribution to the separation of molecular biological subtypes of breast cancer by amino acid profile was made by Gly (*r* = 0.8766), Phe (*r* = 0.8691), Ala (*r* = 0.8410), Tyr (*r* = 0.8227), Leu + Ile (*r* = 0.7921), and Ser (*r* = 0.7082) for the first principal component, as well as Hyl (*r* = 0.9610), Car (*r* = 0.9370), 1-MH (*r* = 0.9296), Hcit (*r* = 0.8452), Trp (*r* = 0.7925), and t4HYP (*r* = 0.7354) for the second principal component (Figure 5B).

## 4. Discussion

In the course of our study, we identified amino acids, the content of which increases in saliva in breast cancer, both in comparison with healthy controls and in comparison with non-malignant pathologies of the mammary glands. These amino acids include the following: Gly, Leu + Ile, Orn, Phe, Pro, and Tyr (Table 4 and Table 5). For these amino acids, the increase in concentration is practically independent of the clinicopathological and molecular biological characteristics of breast cancer. This fact suggests that these amino acids generally characterize the presence of cancer pathology.

The obtained result is in good agreement with literature data, which show that the content of amino acids in saliva increases in breast cancer [24,25,26,27]. The ratio of the concentration of individual amino acids in saliva in breast cancer compared with healthy controls varies significantly among the authors, which, in our opinion, is due to small sample sizes; a wide range of variations in amino acid content, even in normal conditions; as well as the different structure of the study groups both by stage and by molecular biological subtypes of breast cancer.

We found a statistically significant increase in the Glu content and a decrease in the Gln content in saliva in breast cancer only in comparison with healthy controls (Table 4). The dysregulation of glucose metabolism, especially the shift from oxidative phosphorylation to aerobic glycolysis, also known as the Warburg effect, has been included among the hallmarks of cancer [31]. Glucose metabolism has been shown to be an important event during the initiation and progression of breast cancer [32]. Kou et al. showed that Gln was significantly more consumed by breast cancer cells, whereas Glu and Pro were most released into the media by breast cancer cells [33]. It is known that Gln performs several functions in tumor cells: Gln is an intermediate metabolite for the synthesis of nucleotides and non-essential amino acids and allows for the uptake of other essential amino acids, while Gln is removed from the cell in exchange for the uptake of another amino acid; Gln plays a role in the regeneration of intermediate metabolites of the tricarboxylic acid cycle [34], and it is also important for the synthesis of glutathione [35]. Glutaminase, an enzyme that converts Gln to Glu, is overexpressed in breast cancer, especially in TNBC tumors compared to HER2 and luminal subtypes [36]. Exogenous glutamine is essential for the survival of TNBC cells [37]. Luminal tumors are less dependent on exogenous Gln, not so much because of their lower proliferation, but because luminal cells themselves can synthesize the amino acid by expressing the enzyme glutamine synthetase [38]. HER2+ tumors have a glycolytic phenotype [39], as HER2 promotes glucose utilization [40]. For example, it is known that the inhibition of HER2 leads to low levels of cell proliferation due to a depletion of hexokinase-2 [41].

We have shown that the level of Gln in saliva changes unevenly for different molecular biological subtypes of breast cancer. Thus, for the non-luminal, luminal A, and luminal B HER2-negative breast cancer subgroups, the Gln content decreases compared to the healthy controls, while for the luminal B HER2-positive and TNBC subgroups, it increases (Table 6). It is known that high Gln activity has been detected in HER2-type breast cancer [36], suggesting that Gln dependence is increased in proliferative subtypes of breast cancer [42]. Gln metabolism genes are significantly activated in both epithelial and stromal cells of breast cancer tissues, indicating the role of Gln metabolism in the growth and metastasis of breast cancer [43]. It should be noted that, according to literature data, both the concentrations of Glu and Gln increase in saliva [24,25,26,27]. This once again emphasizes the need to take into account the molecular biological subtype of breast cancer when planning experiments and forming groups, as well as when interpreting the results [44]. In general, we have shown, for the first time, the differences in the amino acid profile of saliva in different molecular biological subtypes of breast cancer. Previously, Murata et al. determined the metabolic profile of saliva for four subtypes of breast cancer [27]. However, differences between molecular biological subtypes of breast cancer are characterized by metabolites other than amino acids.

Gly and Ser are well-known and classical metabolites of glycolysis, which are formed from the intermediate 3-phosphoglycerate [45]. It has been shown that the content of these amino acids also increases in the luminal B HER2-positive and TNBC subgroup (Table 6). Moreover, unlike Glu and Gln, the Gly content in saliva increases both in comparison with healthy controls and in comparison with non-malignant pathologies of the mammary glands.

Similar patterns are shown for Leu + Ile and Pro. In breast cancer, the importance and necessity of the metabolic reprogramming of branched-chain amino acids (BCAAs) was recently highlighted by Zhan et al., who showed that BCAA transaminase 1 promotes breast cancer cell growth by improving mTOR-mediated mitochondrial biogenesis and function [46]. Since cancer cells have high energy requirements, and due to the Warburg effect, glucose is not a sufficient energy source, and cells require energy through the degradation of proteins (mainly collagen), providing, among other things, proline as a substrate for PRODH/POX, which leads to the formation of ATP or the generation of reactive oxygen species [47].

An increased expression of aromatic amino acids in breast cancer has become evident in a variety of recent studies [48]. We also showed an increase in the concentrations of Phe and Tyr in breast cancer, but for Trp, the relationship is ambiguous. In particular, Trp content increases for the luminal B HER2-positive and TNBC subgroups relative to the healthy controls, while for other molecular biological subtypes, it decreases. A study highlighted the importance of the kynurenine pathway in mediating tumor immune evasion, revealing a dysregulation of the kynurenine pathway in the HER2-positive and TNBC subtypes of breast cancer [49]. We have previously shown that Trp can be considered as a marker of TNBC [50].

Increased levels of Arg, Cit, Asp, and Asn have been shown for TNBC [51]. Kanaan et al. showed that TNBC has increased levels of tumor-derived Arg and its intermediates, including Cit [52]. These data suggest changes in multiple pathways associated with a higher amino acid uptake and protein catabolism. Cit and other components of the urea cycle increased significantly, leading to increased pro-inflammatory signaling. Yamashita et al. analyzed metabolites associated with the urea cycle and observed significant increases in Asp, Arg, and Cit levels in TNBC tissue samples [53].

Compared with non-malignant pathologies of the mammary glands, an increase in Asp content and a decrease in Asn content in breast cancer were shown (Table 5). It can be assumed that these amino acids are responsible for differentiating malignant and benign pathologies of the mammary glands from each other. Asn is known to significantly influence breast cancer progression, including promoting tumor metastasis [54], which has led to the use of the bacterial enzyme L-asparaginase to limit the availability of asparagine [55,56].

It is interesting to note that the decision tree structure for classifying the BC, BBL, and HC subgroups does not include amino acids that differ significantly between groups other than Pro. The first branch of the decision tree includes Car (Figure 3). To date, the important biological functions of Car have been established, in particular, the ability to exhibit antioxidant properties aimed at suppressing free radical reactions by interacting with reactive oxygen species [57]. Car is known to suppress the proliferation of tumor cells, including breast cancer cells [58,59]. Overall, the resulting decision tree has a sensitivity of 85.3% and a specificity of 72.0%, which is comparable to the data obtained previously. Thus, for the SFAA index, including Pro, Thr, and His, sensitivity was 88.2% and specificity was 85.7% [25]. Murata et al. showed an AUC of 0.912 (0.838–0.961) [27]; however, the classification model includes not only amino acids but also other metabolites, as well as in the Sugimoto et al. model with an AUC of 0.973 (0.881) [24]. The values we obtained are somewhat lower, but we carried out the classification into three classes simultaneously, which gives a qualitatively new result. It should be emphasized that in 3 out of 4 studies, the number of patients in the sample was 30 people or less [60]. It is this fact, in view of the high heterogeneity of breast cancer, that can explain most of the discrepancies in the list of saliva indicators significant for classification and that places emphasis on the correct formation of the sample with the obligatory indication of the molecular biological subtype of breast cancer.

We have shown that the amino acid profiles of luminal A and luminal B HER2-negative breast cancer are almost identical; in the factor diagram of the principal component analysis, these groups completely overlap with each other (Figure 5). This fact indicates the similarity of the molecular biological characteristics of these subgroups and confirms the possibility of considering these types of cancer as a single group. The subgroups of luminal B HER2-positive and TNBC should be considered as separate species.

Limitations of this study are primarily related to the small number of patients in the non-luminal, luminal B HER2-positive, and TNBC subgroups, which is due to their lower occurrence in the population. Limitations include constructing a decision tree without taking into account the molecular biological subtype of the tumor, which we will do later when expanding the number of patients in each subgroup.

## 5. Conclusions

The amino acid profile of saliva was determined in breast cancer, benign breast pathologies, and healthy controls. It was established that the content of amino acids in saliva in breast cancer increases, with the exception of Gln, Sar, and Thr. It was shown that an increase in Glu concentration and a decrease in Gln concentration in breast cancer is observed only in comparison with healthy controls, while an increase in Asp content and a decrease in Asn content in breast cancer is observed only in comparison with benign breast pathologies. It was shown that changes in the amino acid profile depend on the molecular biological characteristics of the tumor. The most significant differences were found for the luminal B HER2-positive and TNBC subgroups. We proposed the construction of a decision tree for the classification of samples of breast cancer, non-malignant breast pathologies, and healthy controls with a sensitivity of 85.3%, which shows the potential of using the amino acid profile of saliva for the diagnosis of breast cancer, including in the early stages.

## Figures and Tables

**Table 1:** Characteristics of the study group.

Feature		Breast Cancer, n = 116
**Clinical Stage**		
	Stage IA + IB	37
	Stage IIA + IIB	43
	Stage IIIA + IIIB	22
	Stage IIIC + IV	14
**Lymph node status**		
	N_0_	60
	N_1–3_	56
**Subtype**		
	Luminal A-like	40
	Luminal B-like (HER2+)	15
	Luminal B-like (HER2-)	35
	HER2-enriched (Non-Lum)	12
	Triple-negative	14
**HER2 status**		
	HER2-negative	28
	HER2-positive	88
**Estrogen (ER) status**		
	ER-negative	26
	ER-positive	90
**Progesterone (PR) status**		
	PR-negative	46
	PR-positive	70
**Degree of differentiation (G)**		
	G I + II	74
	G III	42
**Ki-67**		
	<20%	59
	>20%	57

**Table 2:** Concentration of amino acids in saliva (nmol/mL).

AAs	Breast Cancer, n = 116	Breast Benign Lesion, n = 24	Healthy Control, n = 25	Kruskal–Wallis Test; *p*-Value
**1-MH**	42.09 [39.13; 100.3]	39.97 [39.21; 100.3]	100.1 [39.25; 100.5]	0.9043; 0.6363
**GABA**	5.28 [4.66; 6.31]	5.05 [4.60; 5.98]	5.57 [4.72; 6.97]	1.076; 0.5838
**Hyl**	82.55 [41.98; 84.27]	72.11 [44.84; 83.92]	83.16 [43.59; 84.01]	0.3879; 0.8237
**Ala**	95.34 [73.80; 120.8]	85.85 [68.09; 108.6]	83.82 [77.30; 133.5]	0.9605; 0.6186
**Arg**	25.63 [15.41; 41.06]	21.37 [15.92; 24.29]	21.68 [17.44; 29.43]	1.465; 0.4807
**Asn**	9.02 [8.30; 10.95]	10.57 [7.88; 12.62]	8.56 [8.25; 14.35]	0.3002; 0.8606
**Asp**	17.43 [10.01; 22.57]	9.57 [8.20; 13.22]	12.42 [7.80; 21.79]	7.920; 0.0191 *
**Car**	35.43 [28.19; 38.38]	36.19 [27.44; 38.12]	34.97 [27.16; 39.18]	0.0979; 0.9522
**Cit**	12.13 [7.35; 17.99]	11.71 [6.17; 14.71]	11.20 [6.92; 23.73]	0.7476; 0.6881
**Glu**	77.92 [50.94; 102.9]	74.53 [51.77; 121.3]	59.18 [44.52; 80.64]	4.323; 0.1151
**Gln**	238.8 [104.8; 412.8]	180.6 [114.5; 439.6]	438.76 [163.7; 638.4]	4.319; 0.1154
**Gly**	257.7 [163.7; 378.7]	160.1 [144.8; 206.4]	186.95 [141.7; 305.6]	7.174; 0.0277 *
**His**	65.82 [57.38; 83.16]	58.90 [55.42; 64.77]	66.12 [55.96; 96.48]	5.662; 0.0589
**HCit**	56.37 [53.24; 57.83]	55.03 [52.63; 58.87]	57.38 [52.64; 61.26]	0.5507; 0.7593
**Leu + Ile**	79.02 [34.42; 110.7]	24.50 [15.76; 32.77]	37.21 [14.17; 68.03]	11.59; 0.0031 *
**Met**	29.79 [24.07; 35.37]	29.99 [27.49; 31.07]	29.86 [28.60; 33.02]	0.8095; 0.6671
**Orn**	50.21 [29.16; 87.71]	25.92 [19.62; 44.20]	33.63 [20.45; 46.21]	16.35; 0.0003 *
**Phe**	54.49 [42.66; 66.64]	34.88 [26.79; 42.23]	39.20 [30.79; 62.09]	21.95; 0.0000 *
**Pro**	122.4 [88.58; 172.6]	73.52 [59.84; 112.3]	74.96 [63.51; 189.41]	14.47; 0.0007 *
**Sar**	47.34 [43.31; 55.20]	52.70 [43.31; 55.79]	50.61 [43.64; 64.01]	1.798; 0.4071
**Ser**	58.51 [47.23; 72.63]	59.22 [39.96; 73.76]	52.19 [40.09; 74.29]	0.7530; 0.6862
**Thr**	193.0 [176.8; 229.9]	242.1 [198.6; 258.0]	224.2 [192.5; 266.0]	4.437; 0.1088 *
**t4HYP**	47.46 [46.94; 48.33]	47.41 [47.31; 47.66]	48.06 [46.98; 51.22]	0.5976; 0.7417
**Trp**	46.42 [30.78; 50.14]	44.58 [26.29; 48.15]	47.43 [27.64; 53.27]	1.029; 0.5979
**Tyr**	145.4 [100.9; 202.7]	96.40 [60.95; 112.9]	94.85 [72.96; 170.0]	16.38; 0.0003 *
**Val**	709.0 [408.9; 1041.0]	676.2 [551.3; 774.2]	557.1 [289.6; 944.9]	1.281; 0.5269

Note: *—differences between the three groups are statistically significant, *p* < 0.05.

**Table 3:** Classification matrix to the classes “Breast cancer (BC)/Breast Benign Lesion (BBL)/Healthy Control (HC)”, according to AA values.

	Observed	Predicted BC	Predicted HC	Predicted BBL	Row Total
Number	BC	99	11	6	116
Row Percentage		85.34%	9.48%	5.17%	
Number	HC	2	18	5	25
Row Percentage		8.00%	72.00%	20.00%	
Number	BBL	2	4	18	24
Row Percentage		8.33%	16.67%	75.00%	

**Table 4:** Relative changes in amino acid concentrations compared to healthy controls (%).

AAs	Stage		Lymph Node Status		HER2 Status		ER Status		PR Status		Degree of Differentiation		Ki-67 Expression	
	I + II	III + IV	N0	N1–3	(−)	(+)	(−)	(+)	(−)	(+)	I + II	III	Low	High
**1-MH**	−56	−60	0	−60	−59	−30	0	−59	0	−60	−60	0	−60	0
**GABA**	−7	2	−7	2	−6	3	−4	−6	−3	−9	−6	−4	−7	−4
**Hyl**	−7	0	0	−33	−22	1	0	−22	0	−39	−37	0	−37	0
**Ala**	13	14	13	14	10	24	7	14	13	15	16	6	16	8
**Arg**	20	7	20	12	19	8	23	18	8	21	20	5	18	15
**Asn**	6	−3	5	5	5	NA	69	2	28	4	0	110	0	110
**Asp**	35	48	34	49	33	65	43	38	41	40	40	40	37	43
**Car**	1	1	2	−5	0	14	8	1	5	−9	−5	3	−8	3
**Cit**	11	8	11	8	2	23	5	10	−3	20	20	−20	20	1
**Glu**	33	22	30	35	33	31	29	34	35	30	44	13	46	15
**Gln**	−60	−40	−60	−42	−52	−42	−42	−69	−49	−37	−61	−40	−68	−40
**Gly**	40	38	29	47	28	60	55	29	43	29	42	32	49	37
**His**	−1	1	−2	1	−2	16	1	−2	−2	1	5	−3	0	−1
**Hcit**	−3	−1	−2	−1	−3	−1	−1	−4	−1	−5	−4	−1	−4	−1
**Leu+Ile**	105	138	92	115	92	155	114	112	114	92	114	109	111	114
**Met**	−1	6	0	5	1	−9	4	0	3	−1	2	0	−1	6
**Orn**	48	50	47	50	35	103	51	44	52	38	54	28	44	51
**Phe**	37	46	33	44	34	47	43	36	44	34	46	32	44	36
**Pro**	68	57	61	70	62	81	71	63	60	67	64	60	71	57
**Sar**	−6	−9	−6	−8	−6	−8	−3	−7	−4	−8	−6	−3	−6	−7
**Ser**	13	9	11	13	9	19	11	13	13	10	18	5	19	6
**Thr**	−17	9	−17	−3	−14	3	−11	−17	−16	−12	−12	−18	−13	−15
**t4HYP**	−2	1	−1	0	−1	NA	1	−2	0	−1	−2	0	−2	0
**Trp**	−5	−2	0	−7	−7	6	2	−7	−1	−6	−7	0	−8	−1
**Tyr**	54	53	52	54	42	70	59	52	55	42	56	33	57	44
**Val**	12	36	10	32	11	41	47	7	30	12	29	9	30	9

Note: Red shows a decrease in amino acid concentration, and blue shows an increase. The intensity of the color is proportional to the degree of change in concentration. NA—not assessed.

**Table 5:** Relative changes in amino acid concentrations compared to BBL (%).

AAs	Stage		Lymph Node Status		HER2 Status		ER Status		PR Status		Degree of Differentiation		Ki-67 Expression	
	I + II	III + IV	N0	N1–3	(−)	(+)	(−)	(+)	(−)	(+)	I + II	III	Low	High
**1-MH**	10	0	150	1	3	74	151	3	151	−1	0	151	0	150
**GABA**	3	13	3	13	4	13	6	4	7	1	3	6	3	6
**Hyl**	7	15	15	−22	−10	16	15	−10	15	−30	−27	15	−27	15
**Ala**	11	11	11	11	7	21	5	12	10	12	13	3	13	6
**Arg**	22	8	22	13	20	10	24	20	10	23	22	6	20	16
**Asn**	−14	−21	−15	−15	−15	NA	37	−17	4	−16	−19	70	−19	70
**Asp**	75	92	74	94	73	115	86	79	83	82	82	82	77	86
**Car**	−2	−2	−2	−8	−3	10	4	−2	1	−12	−8	0	−11	−1
**Cit**	7	3	6	4	−3	17	0	6	−7	14	14	−23	15	−4
**Glu**	6	−3	3	7	5	4	2	6	7	3	14	−11	16	−8
**Gln**	−4	46	−4	42	16	41	41	−24	23	52	−4	46	−21	46
**Gly**	63	61	51	71	49	87	81	51	67	51	66	54	74	60
**His**	11	13	10	13	9	30	14	10	10	14	18	9	13	11
**Hcit**	1	3	2	3	1	3	3	0	3	−1	0	3	0	3
**Leu + Ile**	211	261	192	226	191	288	225	223	226	191	225	218	220	226
**Met**	−1	5	−1	5	0	−10	4	−1	3	−1	2	−1	−1	5
**Orn**	92	94	90	94	75	163	96	87	98	80	100	65	87	96
**Phe**	54	64	49	62	50	65	61	53	62	50	64	48	62	52
**Pro**	71	60	64	73	65	85	74	66	63	70	67	63	74	60
**Sar**	−10	−13	−9	−12	−10	−11	−7	−10	−8	−12	−10	−7	−10	−10
**Ser**	−1	−4	−2	−1	−4	5	−2	−1	−1	−3	4	−8	5	−6
**Thr**	−23	1	−23	−11	−21	−4	−17	−23	−22	−19	−19	−24	−19	−21
**t4HYP**	0	2	0	1	0	2	2	0	1	0	0	1	−1	1
**Trp**	1	4	7	−1	−1	12	8	−1	6	0	−1	7	−2	6
**Tyr**	51	50	50	51	40	67	57	49	53	40	54	31	54	42
**Val**	−8	12	−10	8	−9	16	21	−12	7	−8	6	−10	7	−10

Note: Red shows a decrease in amino acid concentration, and blue shows an increase. The intensity of the color is proportional to the degree of change in concentration. NA—not assessed.

**Table 6:** Relative changes in amino acid concentrations compared to healthy controls for different molecular biological subtypes of breast cancer (%).

AAs	Lum A	Lum B (−)	Lum B (+)	Non-Lum	TNBC
**1-MH**	−60	0	−61	0	0
**GABA**	−9	−7	6	−9	2
**Hyl**	−46	−1	−18	0	2
**Ala**	15	4	48	−4	18
**Arg**	20	19	−21	1	85
**Asn**	0	NA	NA	NA	69
**Asp**	35	29	84	22	68
**Car**	−12	2	20	9	37
**Cit**	20	−8	38	−32	19
**Glu**	45	15	34	8	29
**Gln**	−71	−59	51	−44	45
**Gly**	24	20	93	32	73
**His**	−1	−5	19	−9	2
**Hcit**	−5	−1	−4	−2	2
**Leu + Ile**	82	83	174	90	136
**Met**	−1	4	−28	4	25
**Orn**	35	27	163	11	99
**Phe**	34	33	54	44	37
**Pro**	63	57	89	61	73
**Sar**	−8	−6	−5	−4	6
**Ser**	7	13	25	13	15
**Thr**	−13	−20	16	−16	42
**t4HYP**	−2	−1	NA	0	1
**Trp**	−31	−5	25	0	27
**Tyr**	53	39	76	2	62
**Val**	10	6	34	52	57

Note: Red shows a decrease in amino acid concentration, and blue shows an increase. The intensity of the color is proportional to the degree of change in concentration. NA—not assessed.

## Data Availability

The raw data supporting the conclusions of this article will be made available by the authors on request.

## Supplementary Materials

The following supporting information can be downloaded at: https://www.mdpi.com/article/10.3390/cimb46060336/s1, Table S1: Salivary amino acid concentrations in breast cancer depending on the stage; Table S2: Salivary concentrations of amino acids in breast cancer depending on the presence of metastases in the lymph nodes; Table S3: Salivary concentrations of amino acids in breast cancer depending on the degree of differentiation; Table S4: Salivary amino acid concentrations in breast cancer depending on HER2 receptor expression status; Table S5: Salivary amino acid concentrations in breast cancer depending on ER receptor expression status; Table S6: Salivary amino acid concentrations in breast cancer depending on PR receptor expression status; Table S7: Salivary amino acid concentrations in breast cancer depending on the proliferative activity index; Table S8: Salivary amino acid concentrations depending on the molecular biological subtype of breast cancer.

## Abbreviations

AAs—amino acids; t4HYP—Trans-4-hydroxy-L-proline; Hyl —5-Hydroxy-L-lysine; GABA—gamma-aminobutyric acid; 1-MH—1-MethyHistidine; HCit—homocitrulline; Car—carnosine; Sar—sarcosine; Ala—alanine; Arg—arginine; Asn—asparagine; Asp—asparagic acid; Cys—cysteine; Gln—glutamine; Glu—glutamic acid; Gly—glycine; His—hystidine; Ile—isoleucine; Leu—leucine; Lys—lysine; Met—methionine; Phe—phenylalanine; Pro—proline; Ser—serine; Thr—threonine; Trp—tryptophan; Tyr—tyrosine; Val—valine; BC—breast cancer; BBL—breast benign lesion; HC—healthy control; AUC—area under the curve.
