# Supplementary material for: Free Salivary Amino Acid Profile in Breast Cancer: Clinicopathological and Molecular Biological Features
Source: Curr Issues Mol Biol. 2024 Jun 5;46(6):5614–31. doi: 10.3390/cimb46060336 (PMC11202888; doi:10.3390/cimb46060336)
Supplement: Supplementary file 1 [file cimb-46-00336-s001.zip › cimb-3037498-supplementary.pdf]

Table S1. Salivary amino acid concentrations in breast cancer depending on stage, nmol/mL

AAs	St I + II, n=80	p-value St I+II vs. HC	St III + IV, n=36	p-value St III+IV vs. HC	p-value St I+II vs. III+IV
1-MH	44.09 [39.17; 100.41]	0.4880	39.80 [39.04; 100.26]	0.2265	0.5030
GABA	5.21 [4.67; 6.16]	0.2720	5.70 [4.60; 6.65]	0.5674	0.5115
Hyl	76.98 [41.60; 84.27]	0.5095	82.74 [41.98; 84.58]	0.7311	0.8480
Ala	95.07 [73.37; 121.49]	0.8332	95.34 [74.98; 117.34]	0.7693	0.6761
Arg	26.11 [16.43; 41.06]	0.6841	23.09 [12.26; 40.79]	0.5067	0.2809
Asn	9.12 [8.52; 10.15]	0.4623	8.32 [7.47; 18.00]	0.6831	0.5403
Asp	16.74 [9.30; 22.53]	0.3769	18.39 [15.18; 23.21]	0.0541	0.2367
Car	35.43 [28.32; 38.17]	0.8464	35.41 [27.53; 43.86]	0.7456	0.7981
Cit	12.48 [7.35; 17.74]	0.9640	12.04 [7.29; 20.12]	0.8259	0.8814
Glu	78.83 [53.85; 102.88]	0.0280	72.42 [47.78; 103.77]	0.2180	0.5077
Gln	173.35 [89.42; 371.02]	0.0155	263.19 [196.45; 661.24]	0.7359	0.0919
Gly	261.03 [156.97; 384.61]	0.2559	257.70 [177.67; 327.74]	0.1636	0.7883
His	65.21 [57.32; 82.43]	0.7180	66.60 [57.38; 86.32]	0.7581	0.9429
Hcit	55.59 [53.24; 57.82]	0.5103	56.80 [53.27; 58.11]	0.6394	0.6756
Leu	76.17 [21.98; 107.31]	0.1905	88.48 [63.56; 113.92]	0.0204	0.1741
Met	29.64 [23.27; 35.22]	0.3136	31.60 [24.07; 35.37]	0.7458	0.6014
Orn	49.65 [28.29; 88.17]	0.0128	50.31 [31.20; 86.37]	0.0138	0.6981
Phe	53.78 [41.88; 63.86]	0.1181	57.21 [43.57; 70.92]	0.0352	0.2166
Pro	125.60 [91.50; 162.91]	0.0664	117.58 [84.38; 238.69]	0.0646	0.9192
Sar	47.45 [43.70; 54.93]	0.2633	46.07 [43.25; 56.33]	0.2515	0.7791
Ser	58.84 [47.23; 72.54]	0.5176	56.79 [46.55; 72.63]	0.4115	0.9905
Thr	185.23 [176.19; 202.14]	0.0404	245.17 [215.51; 348.09]	0.4795	0.0195
t4HYP	47.32 [46.81; 47.99]	0.1722	48.33 [47.94; 49.03]	0.6606	0.0417
Trp	44.88 [30.78; 49.62]	0.4701	46.45 [26.10; 54.13]	0.8153	0.7686
Tyr	145.92 [101.47; 205.89]	0.0197	144.96 [100.31; 172.54]	0.0417	0.9060
Val	623.01 [411.42; 1027.93]	0.3473	758.04 [408.86; 1041.30]	0.3798	0.8357

Table S2. Salivary concentrations of amino acids in breast cancer depending on the presence of metastases in the lymph nodes, nmol/mL

AAs	N0, n=60	p-value N0 vs. HC	N1-3, n=56	p-value N1-3 vs. HC	p-value N0 vs. N1-3
1-MH	100.12 [39.24; 100.44]	0.7690	40.17 [39.07; 100.20]	0.1392	0.2843
GABA	5.19 [4.68; 5.91]	0.1962	5.70 [4.62; 7.07]	0.6021	0.2716
Hyl	82.78 [41.53; 84.89]	0.7630	56.09 [42.29; 83.50]	0.3851	0.5736
Ala	95.07 [72.54; 119.84]	0.9232	95.34 [75.00; 120.76]	0.9837	0.8684
Arg	26.00 [16.43; 41.03]	0.7598	24.24 [12.88; 41.58]	0.8063	0.6666
Asn	9.02 [8.30; 9.35]	0.6606	8.98 [8.32; 10.95]	0.7963	1.0000
Asp	16.63 [9.98; 21.65]	0.4379	18.52 [10.90; 24.96]	0.0901	0.1168
Car	35.55 [28.19; 39.12]	0.7625	33.36 [27.91; 37.86]	0.8814	0.7175
Cit	12.46 [7.14; 17.42]	0.9002	12.13 [7.45; 20.12]	0.9348	0.8553
Glu	76.87 [57.67; 99.97]	0.0339	79.61 [48.91; 109.77]	0.1107	0.9076
Gln	173.35 [86.00; 375.56]	0.0304	256.64 [134.89; 412.79]	0.2342	0.2694
Gly	241.46 [148.32; 384.61]	0.3348	274.25 [180.75; 367.85]	0.1355	0.6190
His	64.74 [57.32; 82.43]	0.6783	66.75 [57.38; 84.56]	0.7904	0.6949
Hcit	55.98 [53.34; 57.83]	0.6341	56.65 [53.08; 57.94]	0.4675	0.8885
Leu	71.49 [16.97; 104.82]	0.2742	79.84 [46.62; 112.00]	0.0220	0.1692
Met	29.74 [22.12; 34.21]	0.3542	31.37 [24.07; 36.89]	0.5342	0.6586
Orn	49.33 [28.10; 82.37]	0.0139	50.31 [31.49; 88.17]	0.0126	0.7278
Phe	52.11 [42.31; 63.57]	0.0785	56.44 [42.66; 71.00]	0.0844	0.2367
Pro	120.93 [88.58; 150.92]	0.1094	127.22 [89.30; 188.72]	0.0370	0.5038
Sar	47.82 [43.70; 55.86]	0.3571	46.38 [43.25; 54.20]	0.1790	0.5581
Ser	58.16 [47.04; 73.77]	0.4814	58.83 [48.58; 71.82]	0.4805	0.9252
Thr	185.23 [175.31; 197.29]	0.0494	216.41 [190.19; 245.17]	0.7223	0.0621
t4HYP	47.42 [47.05; 48.02]	0.3159	47.94 [46.48; 49.03]	0.6434	0.6121
Trp	47.62 [38.96; 52.53]	0.9310	44.30 [26.41; 47.52]	0.2342	0.2235
Tyr	144.50 [101.78; 185.74]	0.0186	145.69 [96.56; 216.13]	0.0357	0.7799
Val	611.61 [393.01; 1041.01]	0.4383	733.24 [411.42; 1040.46]	0.2797	0.6890

Table S3. Salivary concentrations of amino acids in breast cancer depending on the degree of differentiation, nmol/mL

AAs	G I-II, n=74	G I-II vs. HC	G III, n=42	G III vs. HC	G I-II vs. G III
1-MH	39.87 [39.07; 100.29]	0.1381	100.15 [39.27; 100.50]	0.9624	0.1794
GABA	5.22 [4.66; 6.43]	0.3258	5.33 [4.67; 6.19]	0.4141	0.8587
Hyl	52.30 [41.98; 84.51]	0.3740	83.01 [41.26; 84.27]	0.9167	0.6815
Ala	97.00 [74.68; 120.29]	0.8975	88.56 [73.53; 123.39]	0.7557	0.5541
Arg	26.04 [15.50; 45.60]	0.6971	22.73 [12.95; 38.38]	0.5960	0.3641
Asn	8.53 [8.06; 9.12]	0.7527	18.00 [10.95; 72.75]	0.1025	0.0143
Asp	17.44 [10.32; 22.52]	0.2167	17.43 [9.88; 22.89]	0.2223	0.9033
Car	33.36 [27.91; 38.07]	0.9192	36.09 [28.32; 39.57]	0.6784	0.4236
Cit	13.39 [7.82; 20.11]	0.7596	8.96 [6.94; 16.73]	0.4443	0.1680
Glu	85.19 [62.20; 112.12]	0.0102	66.70 [47.54; 85.06]	0.4599	0.0101
Gln	173.26 [89.83; 394.18]	0.0226	263.42 [125.05; 545.36]	0.3507	0.2855
Gly	265.71 [168.41; 394.72]	0.1240	247.24 [140.85; 322.22]	0.4758	0.2126
His	69.66 [57.68; 85.75]	0.9230	63.93 [54.51; 79.86]	0.4599	0.2853
Hcit	55.27 [53.24; 57.32]	0.4366	56.65 [53.04; 58.58]	0.7340	0.6224
Leu	79.59 [36.96; 111.29]	0.0682	77.84 [25.80; 100.35]	0.1813	0.5616
Met	30.45 [24.07; 36.89]	0.3753	29.77 [22.12; 32.46]	0.5154	0.8679
Orn	51.96 [29.89; 87.96]	0.0056	42.89 [28.44; 78.15]	0.0445	0.3641
Phe	57.17 [44.13; 69.85]	0.0344	51.58 [39.58; 57.48]	0.2436	0.0499
Pro	122.93 [89.77; 188.63]	0.0377	119.60 [85.43; 148.65]	0.1465	0.4763
Sar	47.34 [43.36; 55.21]	0.2441	49.15 [42.62; 55.18]	0.2863	0.9450
Ser	61.65 [49.06; 72.88]	0.3585	54.66 [46.02; 72.17]	0.7166	0.3491
Thr	197.29 [176.19; 229.86]	0.1928	183.45 [180.60; 202.14]	0.2703	0.9607
t4HYP	47.21 [46.81; 47.38]	0.0914	48.06 [47.94; 48.67]	0.8474	0.0071
Trp	44.02 [30.03; 50.56]	0.3942	47.62 [44.12; 49.88]	0.8744	0.3098
Tyr	148.27 [101.90; 212.17]	0.0079	126.40 [93.53; 165.90]	0.1180	0.2057
Val	716.85 [446.66; 1040.46]	0.2772	607.41 [367.06; 1041.01]	0.5222	0.5667

Table S4. Salivary amino acid concentrations in breast cancer depending on HER2 receptor expression status, nmol/mL

AAs	HER2 (-), n=88	HER2 (-) vs. HC	HER2 (+), n=28	HER2 (+) vs. HC	HER2 (-) vs. HER2 (+)
1-MH	41.13 [39.17; 100.34]	0.4162	69.61 [38.94; 100.31]	0.2134	0.4176
GABA	5.22 [4.66; 6.34]	0.2963	5.72 [4.55; 6.31]	0.5640	0.7266
Hyl	65.06 [41.60; 84.27]	0.4123	83.61 [51.15; 84.58]	0.6951	0.3497
Ala	91.85 [72.21; 116.27]	0.7609	104.19 [84.38; 129.45]	0.4363	0.0925
Arg	25.71 [15.97; 41.06]	0.7256	23.45 [12.29; 46.39]	0.5089	0.4814
Asn	9.02 [8.30; 10.95]	0.6797		1.0000	1.0000
Asp	16.51 [9.30; 21.65]	0.3271	20.55 [17.16; 23.22]	0.0327	0.0326
Car	34.95 [28.19; 37.86]	0.9277	39.98 [27.42; 45.93]	0.5485	0.3827
Cit	11.40 [7.35; 19.11]	0.8302	13.75 [7.55; 17.89]	0.6938	0.5794
Glu	78.56 [49.76; 107.10]	0.0638	77.23 [57.99; 100.85]	0.0684	0.8846
Gln	209.79 [89.83; 394.18]	0.0338	255.13 [238.78; 611.84]	0.8153	0.2798
Gly	239.10 [145.98; 370.94]	0.3910	299.30 [218.39; 395.03]	0.0261	0.0600
His	64.50 [55.98; 80.95]	0.5519	76.64 [62.77; 87.04]	0.6145	0.1118
Hcit	55.59 [53.19; 57.83]	0.5448	56.65 [53.87; 58.28]	0.5751	0.7814
Leu	71.31 [23.77; 109.81]	0.1827	94.94 [77.38; 117.37]	0.0140	0.0237
Met	30.12 [24.33; 35.62]	0.5200	27.02 [21.09; 35.08]	0.1834	0.5160
Orn	45.33 [28.04; 77.58]	0.0269	68.27 [40.79; 95.42]	0.0006	0.0188
Phe	52.38 [42.04; 63.57]	0.1189	57.72 [52.09; 71.00]	0.0275	0.0393
Pro	121.64 [83.22; 172.63]	0.0778	135.75 [102.13; 220.13]	0.0394	0.2272
Sar	47.34 [43.25; 55.33]	0.2146	46.80 [43.58; 54.07]	0.3454	0.9947
Ser	56.85 [45.59; 71.82]	0.6630	62.15 [52.11; 78.22]	0.1842	0.1892
Thr	192.15 [176.19; 202.97]	0.1279	231.25 [180.60; 259.10]	0.8658	0.3135
t4HYP	47.38 [46.94; 48.06]	0.3220	48.13 [47.94; 48.33]	1.0000	0.3524
Trp	44.06 [30.03; 48.82]	0.2907	50.14 [47.05; 59.20]	0.4176	0.1215
Tyr	134.66 [101.47; 199.42]	0.0257	161.43 [96.56; 205.89]	0.0231	0.3194
Val	618.36 [374.35; 1034.19]	0.5473	783.87 [547.53; 1054.56]	0.1181	0.1674

Table S5. Salivary amino acid concentrations in breast cancer depending on ER receptor expression status, nmol/mL

AAs	ER (-), n=37	ER (-) vs. HC	ER (+), n=79	ER (+) vs. HC	ER(-) vs ER(+)
1-MH	100.19 [39.04; 100.39]	0.5041	41.11 [39.15; 100.32]	0.3653	0.8385
GABA	5.33 [4.70; 5.97]	0.3775	5.23 [4.66; 6.47]	0.3475	0.8334
Hyl	83.01 [43.62; 85.40]	0.9728	65.06 [41.79; 84.25]	0.4138	0.3650
Ala	89.95 [75.32; 123.39]	0.9943	95.92 [72.54; 116.28]	0.9484	0.8893
Arg	26.59 [15.97; 52.78]	0.5361	25.63 [15.41; 38.38]	0.7862	0.1893
Asn	14.48 [10.95; 18.00]	0.2963	8.75 [8.30; 9.22]	0.9233	0.0990
Asp	17.81 [12.42; 23.83]	0.1052	17.16 [9.79; 22.08]	0.3034	0.2959
Car	37.79 [27.63; 45.93]	0.5982	35.40 [28.19; 38.38]	0.9343	0.4788
Cit	11.71 [7.37; 20.11]	0.9371	12.37 [7.34; 17.89]	0.9061	0.9551
Glu	76.19 [48.03; 98.87]	0.1493	79.04 [53.70; 107.55]	0.0361	0.5556
Gln	255.13 [196.54; 466.36]	0.4350	136.60 [86.82; 412.79]	0.0248	0.2733
Gly	289.66 [191.08; 358.93]	0.1453	241.48 [153.58; 379.28]	0.2717	0.5516
His	66.90 [57.53; 84.34]	0.9485	65.06 [57.22; 82.79]	0.6237	0.6849
Hcit	56.86 [53.39; 60.36]	0.9741	54.94 [53.10; 57.72]	0.3586	0.2498
Leu	79.59 [56.69; 126.33]	0.0143	79.02 [18.62; 107.31]	0.2135	0.1354
Met	31.11 [24.42; 41.96]	0.8360	29.74 [23.35; 35.37]	0.2829	0.5284
Orn	50.85 [29.89; 91.45]	0.0169	48.46 [28.14; 86.58]	0.0114	0.6462
Phe	56.07 [44.22; 70.96]	0.0559	53.34 [41.88; 63.86]	0.0972	0.3063
Pro	128.02 [89.77; 151.37]	0.0864	122.14 [87.39; 181.11]	0.0567	0.9693
Sar	49.24 [42.58; 56.96]	0.3837	47.25 [43.36; 54.34]	0.2063	0.5797
Ser	58.12 [50.62; 74.63]	0.2661	58.82 [46.02; 72.17]	0.6237	0.4608
Thr	199.78 [180.60; 231.25]	0.4350	187.01 [176.19; 202.97]	0.1213	0.4970
t4HYP	48.33 [47.94; 48.40]	0.8836	47.32 [46.81; 47.99]	0.2192	0.0641
Trp	48.22 [46.45; 59.31]	0.6954	44.04 [28.22; 48.49]	0.2732	0.0730
Tyr	151.10 [96.08; 222.84]	0.0346	144.02 [101.84; 175.18]	0.0214	0.7460
Val	818.75 [414.09; 1422.26]	0.1855	596.21 [393.01; 946.16]	0.4762	0.1817

Table S6. Salivary amino acid concentrations in breast cancer depending on PR receptor expression status, nmol/mL

AAs	PR (-), n=60	PR (-) vs. HC	PR (+), n=56	PR (+) vs. HC	PR (-) vs. PR (+)
1-MH	100.18 [39.45; 100.43]	0.9249	39.69 [39.08; 100.20]	0.1689	0.1930
GABA	5.42 [4.56; 6.31]	0.3697	5.08 [4.68; 6.32]	0.3469	0.9471
Hyl	83.01 [48.58; 84.21]	0.8841	50.66 [41.41; 84.70]	0.2570	0.2495
Ala	94.58 [74.95; 123.04]	0.8546	95.98 [72.88; 116.27]	0.7825	0.6989
Arg	23.45 [12.80; 44.79]	0.6085	26.18 [16.91; 39.08]	0.5301	0.5160
Asn	10.95 [8.30; 18.00]	0.6831	8.88 [8.07; 9.28]	0.7527	0.4142
Asp	17.48 [10.90; 23.22]	0.1214	17.38 [9.11; 21.65]	0.3747	0.3027
Car	36.62 [28.63; 40.40]	0.3593	31.98 [27.63; 37.72]	0.7210	0.1169
Cit	10.89 [6.88; 17.05]	0.5563	13.39 [7.80; 22.50]	0.6826	0.1759
Glu	79.92 [49.58; 102.88]	0.0562	76.87 [57.67; 103.88]	0.0688	0.8858
Gln	222.94 [121.46; 354.78]	0.1051	275.21 [86.82; 412.79]	0.0652	0.9326
Gly	267.58 [177.54; 407.94]	0.1678	241.46 [156.97; 375.01]	0.2923	0.6268
His	64.88 [57.16; 82.35]	0.6643	66.89 [58.13; 85.67]	0.8062	0.7824
Hcit	56.65 [53.61; 57.95]	0.6270	54.57 [53.14; 57.52]	0.4939	0.3527
Leu	79.82 [46.62; 115.85]	0.0232	71.31 [16.55; 104.82]	0.3451	0.1204
Met	30.82 [22.56; 43.28]	0.7100	29.54 [24.33; 33.89]	0.2243	0.3515
Orn	51.28 [28.25; 93.08]	0.0132	46.54 [30.55; 81.16]	0.0134	0.7612
Phe	56.44 [44.13; 69.85]	0.0366	52.38 [41.88; 66.63]	0.1710	0.1881
Pro	119.60 [88.63; 175.61]	0.0776	124.93 [88.58; 170.69]	0.0559	0.7319
Sar	48.47 [43.35; 56.22]	0.2985	46.59 [43.31; 54.34]	0.2233	0.5507
Ser	58.83 [49.46; 78.25]	0.2715	57.57 [44.91; 71.73]	0.7747	0.3041
Thr	188.61 [176.77; 231.25]	0.1809	197.29 [175.31; 229.86]	0.2831	0.9639
t4HYP	47.94 [46.81; 48.33]	0.4414	47.42 [47.15; 48.06]	0.4239	0.8703
Trp	47.05 [43.63; 50.14]	0.7851	44.74 [26.26;49.69]	0.3861	0.3752
Tyr	147.35 [106.21; 205.89]	0.0133	135.08 [96.76; 199.42]	0.0488	0.4018
Val	726.60 [386.78; 1135.68]	0.3704	623.01 [449.25; 887.38]	0.3471	0.7363

Table S7. Salivary amino acid concentrations in breast cancer depending on the proliferative activity index, nmol/mL

AAs	Ki-67 low, n=57	Ki-67 low vs. HC	Ki-67 high, n=59	Ki-67 high vs. HC	Ki-67 low vs. Ki-67 high
1-MH	40.13 [39.07; 100.39]	0.2497	100.09 [39.23; 100.34]	0.6601	0.5599
GABA	5.20 [4.69; 6.43]	0.5159	5.33 [4.53; 6.19]	0.2384	0.5527
Hyl	52.30 [41.60; 84.51]	0.4317	82.74 [41.98; 84.27]	0.7506	0.8256
Ala	97.22 [74.68; 116.25]	0.9879	90.83 [73.53; 123.39]	0.9260	0.7761
Arg	25.63 [15.58; 44.76]	0.7735	24.82 [12.88; 41.06]	0.8385	0.7260
Asn	8.53 [8.06; 9.12]	0.7527	18.00 [10.95; 72.75]	0.1025	0.0143
Asp	16.98 [9.30; 22.60]	0.3267	17.81 [10.90; 22.04]	0.1419	0.6773
Car	32.10 [27.63; 37.72]	0.8505	35.92 [28.43; 40.40]	0.4893	0.1998
Cit	13.44 [7.60; 18.10]	0.8680	11.28 [7.34; 17.59]	0.7137	0.5093
Glu	86.13 [64.98; 117.88]	0.0052	68.26 [47.54; 89.22]	0.3352	0.0052
Gln	142.48 [86.41; 459.96]	0.0234	263.19 [136.60; 375.56]	0.2453	0.1866
Gly	278.39 [166.92; 394.26]	0.1541	256.35 [146.93; 352.41]	0.3066	0.4952
His	66.29 [57.54; 83.53]	0.7056	65.35 [57.22; 82.79]	0.7580	0.9978
Hcit	55.27 [53.24; 57.94]	0.6050	56.65 [53.04; 57.83]	0.4958	0.8885
Leu	78.50 [16.55; 111.29]	0.2167	79.82 [39.40; 109.81]	0.0420	0.4805
Met	29.64 [24.33; 34.83]	0.2795	31.60 [22.12; 41.96]	0.6214	0.3933
Orn	48.46 [28.06; 88.59]	0.0142	50.85 [30.58; 78.15]	0.0124	0.8273
Phe	56.43 [42.04; 69.34]	0.0600	53.16 [43.45; 60.53]	0.1062	0.3200
Pro	127.90 [91.16; 186.80]	0.0445	117.79 [84.78; 170.10]	0.0953	0.4346
Sar	47.41 [43.58; 54.69]	0.2867	47.20 [43.12; 55.24]	0.2359	0.9406
Ser	62.15 [49.06; 71.73]	0.4776	55.50 [46.88; 78.22]	0.4842	0.8489
Thr	195.37 [176.19; 229.86]	0.1988	190.37 [178.69; 229.44]	0.2481	1.0000
t4HYP	47.16 [27.96; 47.76]	0.1415	47.94 [47.27; 48.67]	0.8044	0.0986
Trp	43.60 [26.26; 49.22]	0.3014	47.05 [44.02; 54.13]	0.9106	0.1669
Tyr	148.93 [101.78; 212.24]	0.0088	136.83 [97.53; 167.40]	0.0622	0.3761
Val	724.22 [449.25; 1040.01]	0.2285	607.41 [335.04; 1041.30]	0.5258	0.4397

Table S8. Salivary amino acid concentrations depending on the molecular biological subtype of breast cancer, nmol/mL

AAs	Lum A, n=40	Lum B(-), n=35	Lum B(+), n=15	Non-Lum, n=12	TNBC, n=14
1-MH	39,74<br>[39,07; 100,2]	100,1<br>[39,31; 100,3]	38,92	100,2<br>[39,04; 100,4]	100,5<br>[40,17; 101,1]
GABA	5,08<br>[4,69; 6,86]	5,20<br>[4,47; 6,09]	5,90<br>[5,00; 8,04]	5,08<br>[4,39; 6,04]	5,67<br>[4,82; 6,73]
Hyl	45,14<br>[41,53; 84,51]	82,64<br>[41,98; 83,88]	67,86<br>[51,15; 84,58]	83,03<br>[83,01; 84,21]	84,58<br>[63,18; 87,74]
Ala	95,98<br>[71,41; 118,3]	87,16<br>[70,02; 112,0]	124,2<br>[95,27; 135,6]	80,14<br>[72,33; 106,9]	98,51<br>[83,84; 147,5]
Arg	25,94<br>[18,61; 39,08]	25,71<br>[15,61; 40,00]	17,09<br>[12,08; 48,00]	21,81<br>[16,31; 36,80]	40,20<br>[12,21; 53,46]
Asn	8,53<br>[8,06; 9,12]	72,75	-	-	14,48<br>[10,95; 18,00]
Asp	16,74<br>[9,11; 22,08]	16,08<br>[9,29; 19,17]	22,81<br>[18,09; 24,98]	15,18<br>[9,02; 19,51]	20,86<br>[16,33; 23,83]
Car	30,69<br>[27,43; 36,34]	35,73<br>[28,75; 37,64]	41,80	38,12<br>[27,42; 45,93]	48,00<br>[33,14; 68,74]
Cit	13,39<br>[7,71; 22,48]	10,29<br>[6,42; 16,73]	15,51<br>[11,71; 20,11]	7,59<br>[6,45; 12,54]	13,34<br>[7,83; 23,61]
Glu	86,00<br>[62,60; 122,41]	68,35<br>[47,83; 106,7]	79,04<br>[74,36; 102,08]	63,69<br>[45,50; 102,26]	76,37<br>[48,03; 98,01]
Gln	127,9<br>[86,8; 507,1]	179,8<br>[86,0; 354,8]	661,2	246,2<br>[196,4; 256,6]	638,0<br>[229,9; 2280,6]
Gly	232,0<br>[150,8; 392,3]	225,1<br>[137,0; 320,3]	360,5<br>[285,0; 526,4]	246,7<br>[192,7; 306,2]	323,8<br>[146,9; 468,8]
His	65,68<br>[55,98; 84,56]	62,84<br>[55,28; 79,86]	78,67<br>[64,42; 87,04]	60,22<br>[53,00; 74,77]	67,40<br>[59,65; 91,41]
Hcit	54,57<br>[53,19; 57,82]	56,53<br>[53,04; 57,83]	55,30<br>[53,87; 56,74]	56,51<br>[54,40; 58,28]	58,71<br>[54,90; 65,24]
Leu	67,84<br>[11,50; 90,33]	68,09<br>[22,14; 110,2]	102,0<br>[87,89; 126,1]	70,78<br>[35,28; 89,22]	87,75<br>[68,18; 129,8]
Met	29,54<br>[24,33; 34,83]	31,03<br>[27,78; 35,37]	21,57<br>[19,63; 36,89]	31,11<br>[28,34; 32,41]	37,21<br>[18,57; 82,80]
Orn	45,33<br>[28,04; 81,16]	42,73<br>[25,57; 76,84]	88,37<br>[64,05; 98,13]	37,48<br>[24,69; 66,58]	66,84<br>[30,58; 91,45]
Phe	52,38<br>[39,79; 69,34]	51,94<br>[39,58; 59,05]	60,53<br>[55,40; 74,81]	56,44<br>[41,28; 64,48]	53,53<br>[44,30; 70,88]
Pro	122,2<br>[88,58; 199,6]	117,4<br>[73,68; 175,2]	141,5<br>[103,7; 222,3]	120,9<br>[88,63; 182,4]	129,4<br>[84,78; 151,4]
Sar	46,34<br>[43,09; 53,63]	47,48<br>[44,04; 55,42]	48,19<br>[44,15; 53,52]	48,73<br>[43,08; 57,40]	53,84<br>[41,80; 59,51]
Ser	55,72<br>[45,42; 70,64]	58,82<br>[45,52; 72,17]	65,08<br>[53,94; 79,09]	59,12<br>[48,72; 73,76]	59,78<br>[47,27; 95,58]
Thr	195,0<br>[176,2; 229,9]	180,1<br>[175,3; 197,3]	259,1	188,6<br>[180,6; 231,2]	318,4<br>[199,8; 437,1]
t4HYP	47,16<br>[9,10; 47,46]	47,50<br>[46,98; 48,33]	-	48,13<br>[47,94; 48,33]	48,40<br>[47,35; 52,92]
Trp	32,57<br>[24,70; 49,69]	44,88<br>[43,98; 47,62]	59,20<br>[54,13; 59,31]	47,52<br>[47,05; 48,22]	60,30<br>[46,45; 92,18]
Tyr	145,1<br>[96,76; 213,4]	131,7<br>[100,3; 161,2]	167,4<br>[155,8; 216,1]	96,56<br>[79,49; 156,3]	153,3<br>[108,6; 226,8]
Val	611,6<br>[429,0; 849,2]	591,8<br>[267,1; 1041,0]	749,0<br>[554,8; 1261,0]	844,5<br>[408,9; 1054,6]	876,1<br>[330,9; 1439,0]
